# Metagenomic exploration of Andaman region of the Indian Ocean

**DOI:** 10.1038/s41598-024-53190-1

**Published:** 2024-02-01

**Authors:** Vishnu Prasoodanan P. K., Sudhir Kumar, Darshan B. Dhakan, Prashant Waiker, Rituja Saxena, Vineet K. Sharma

**Affiliations:** MetaBioSys Group, Department of Biological Sciences, Indian Institute of Science Education and Research Bhopal, Bhopal, India

**Keywords:** Computational biology and bioinformatics, Microbiology

## Abstract

Ocean microbiome is crucial for global biogeochemical cycles and primary productivity. Despite numerous studies investigating the global ocean microbiomes, the microbiome composition of the Andaman region of the Indian Ocean remains largely unexplored. While this region harbors pristine biological diversity, the escalating anthropogenic activities along coastal habitats exert an influence on the microbial ecology and impact the aquatic ecosystems. We investigated the microbiome composition in the coastal waters of the Andaman Islands by 16S rRNA gene amplicon and metagenomic shotgun sequencing approaches and compared it with the Tara Oceans Consortium. In the coastal waters of the Andaman Islands, a significantly higher abundance and diversity of *Synechococcus* species was observed with a higher abundance of photosynthesis pigment-related genes to adapt to variable light conditions and nutrition. In contrast, *Prochlorococcus* species showed higher abundance in open ocean water samples of the Indian Ocean region, with a relatively limited functional diversity. A higher abundance of antibiotic-resistance genes was also noted in the coastal waters region. We also updated the ocean microbiome gene catalog with 93,172 unique genes from the Andaman coastal water microbiome. This study provides valuable insights into the Indian Ocean microbiome and supplements the global marine microbial ecosystem studies.

## Introduction

Oceans harbor diverse life forms that play a crucial role in global biogeochemical fluxes including nitrogen and carbon cycling. Phytoplankton, such as single-celled algae and cyanobacteria, are prevalent in surface waters and contribute significantly to carbon fixation^1^. Similarly, nitrogen input in oceanic ecosystems is primarily facilitated by bacteria such as *Trichodesmium* and *Crocosphaera* (diazotrophs) that convert atmospheric nitrogen into ammonium which is essential for making amino acids and proteins in all forms of life^2^. Oceanic microbes also influence the cycling of essential nutrients like iron, phosphorus, and sulfur^3,4^.

Cyanobacteria, specifically the dominant genera like *Synechococcus* and *Prochlorococcus*, are the primary autotrophic prokaryotes in marine surface waters that enrich the photosynthetic biomass and play a significant role in ecosystem functioning^5^. Even though both are ubiquitous, their abundance varies on local scales and both have adaptations to the local environmental conditions^6–8^. *Synechococcus* species have evolved and accumulated a wide variety of pigmentation that allow them to grow in varying light conditions in well-mixed coastal environments^9^. In contrast, *Prochlorococcus* is perhaps the most abundant photosynthetic organism on earth and can adapt to low nutrient conditions in oligotrophic oceans depleted of macronutrients^8^.

In recent years, next-generation sequencing and integration of multi-omics approaches have advanced the understanding of taxonomic and functional composition of ocean microbiome. Craig Venter pioneered the use of metagenomic sequencing for studying ocean microbial diversity. They employed shotgun sequencing techniques to obtain about 1 Gbp of sequencing data from the Sargasso Sea near Bermuda^10^. Subsequently, the Sorcerer II Global Ocean Sampling (GOS) project conducted sampling along the North American coast, eastern tropical Pacific, and equatorial Indian Ocean, focusing on surface waters^11^. Later on, Beatriz Díez et al. studied the diversity and functional aspects of picocyanobacteria in open waters of the equatorial Indian Ocean^12^. Further studies in the field explored the deep-water samples to understand the effects of factors such as sunlight, pressure, and oxygen levels that vary with depth and their influence on microbial diversity^13–16^.

A large-scale effort for a holistic understanding of marine water communities was conducted through the Tara Oceans expedition, which collected samples from diverse global locations and generated extensive metagenomic sequence data (https://ocean-microbiome.embl.de/companion.html). This expedition covered the Pacific Ocean, the North and South Atlantic Oceans, and the western parts of the Indian Ocean, specifically the Arabian Sea within the Indian Monsoon Gyres Province (MONS) and Northwestern Arabian Sea Upwelling Province (ARAB). The data obtained from this expedition contributed to the construction of a reference metagenomic gene catalog from marine water environments^17^.

To date, the microbiome composition of the eastern part of the Indian Ocean remains largely unexplored. The studies conducted in the Indian Ocean region were either limited to amplicon-based sequencing, which lacks comprehensive functional representation of microbial communities, or their sampling was confined to the equatorial Indian Ocean region^12,14–16^. In addition, the majority of previous studies were focused on the microbiome of open ocean regions. There are a few reports of culture-based exploration in these areas^18^, however, the microbial composition of coastal waters remains largely understudied.

The Andaman and Nicobar Islands region in the Indian Ocean has diverse terrestrial and marine ecosystems like mangroves, coral reefs, beaches, etc. The increasing impact of various human activities in the coastal areas, which primarily contribute to the aquatic food-animal supply, could be a potential source of antibiotic resistance transmission^19,20^. The majority of antibiotics are excreted into the environment and end up in aquatic ecosystems, which serve as a reservoir of antibiotic resistance genes (ARGs)^21–23^. Thus, it is important to explore the microbiome composition and genetic makeup of coastal marine ecological units. This diversity also holds substantial potential for biomedical applications^24,25^.

We carried out a comprehensive study to understand the diversity and functional roles of microbial communities in coastal waters from the Andaman Islands which is a yet unexplored part of the Indian Ocean region. We employed a combined approach of 16S rRNA amplicon and whole metagenomic shotgun sequencing to explore microbiome composition and its functional potential. We also used previously available metagenomic data from the Tara Oceans study for comparative analysis of coastal with open ocean microbiome. This study provides the first compositional and functional characterization of the microbial communities in the coastal water region of the Andaman Sea.

## Methods

### Andaman data description

Marine water samples were collected from Andaman and Nicobar Islands, India located at 11.61 °N, 92.74 °E, and Cape-Comorin, the southernmost point of peninsular India. Water samples ranging from 2 to 3 L were collected using sterile plastic cans/carboys and transported to the laboratory in ice packs. All marine water samples were subjected to filtration with a 1.2 µm pore size membrane to avoid contamination of other micro-eukaryotes and suspended organic matter^26^. Nine samples from six locations at variable distances from the coast of the Andaman islands and one sample from Cape-Comorin were collected for 16S rRNA amplicon sequencing. Additionally, the same six samples from each location in the Andaman region were used for whole metagenome sequencing.

### DNA extraction and sequencing

Coarse particles and debris were removed from the samples by filtering through a 1.2 µm membrane filter. To entrap the prokaryotic cells the filtrate was passed through a 0.22 µm pore-size membrane filter^27^. DNA was extracted from this membrane using the metagenomic DNA isolation kit for water (Epicenter, Wisconsin, USA), according to the manufacturer’s protocol with minor modifications like the addition of 100 µl of 5 M NaCl to 700 µl isopropanol for efficient precipitation of DNA. The DNA pellet was resuspended in 10 mM Tris (pH 8.5) and evaluated on a Geneva nanodrop micro-spectrophotometer (Jenway, Bibby Scientific Limited, U.K) and Qubit HS dsDNA kit (Life technologies, USA). We generated an amplicon library targeting the V3 region of the 16S rRNA gene, using purified DNA as a template. This specific region, along with V4 and V3-V4, has demonstrated high accuracy in discerning taxonomic diversity within bacteria^28,29^. The details of primers used for amplification are shared in supplementary data (Supplementary Table 3). PCR was performed with initial denaturation at 94 °C for 5 min, followed by 35 cycles of denaturation at 94 °C for 30 s, annealing at 69 °C for 30 s, extension at 72 °C for 30 s, and final extension cycle for 5 min at 72 °C. Taq DNA Polymerase (Life technologies, USA) was used and 5% DMSO was added to the master mix to enhance the concentration of amplified product from the metagenomic GC-rich template. The amplified products were evaluated on 2% w/v agarose gel and purified using the Agencourt Ampure XP kit (Beckman Coulter, USA). The libraries were prepared using the Illumina 16S metagenomic library preparation guide. The size of prepared library was estimated on 2100 Bioanalyzer using Bioanalyzer DNA 1000 kit (Agilent, USA) and library concentration was measured with Qubit 2.0 fluorometer using dsDNA HS kit (Life technologies, USA).

Shotgun metagenomics libraries were prepared using the Illumina Nextera XT sample preparation kit (Illumina, USA) following the manufacturer’s protocol. The libraries were evaluated on 2100 Bioanalyzer using Bioanalyzer DNA 1000 kit (Agilent, USA) to estimate the library size. Library was quantified using Qubit dsDNA HS kit (Life technologies, USA) and KAPA SYBR Fast qPCR Master mix, Illumina standards, and primer premix (kappa Biosystems, USA). Paired-end sequencing (150 bp) of both libraries was performed on the Illumina NextSeq500 platform (Illumina, USA).

### Collection of publicly available open ocean microbiome data from Tara Oceans Consortium

Open ocean samples from Tara Oceans Consortium^17^ were retrieved for the comparative analysis of community structure with the coastal microbiome. Three criteria were employed for selecting samples from the Tara Ocean Consortium data, as described in the following text. (i) Sample collected from Marine pelagic biomes of ARAB and MONS were used for the analysis, (ii) Only surface water samples were considered (sampling depth = 5 m) to allow relevant comparisons with our data, (iii) Only samples from size fractions of 0.1–0.22 µm and 0.22–1.6 µm were taken into consideration and size fractions of < 0.22 µm were excluded. Using these criteria, a total of 985,580,126 reads (109,508,902.88 ± 83,144,900.59, mean ± sd) from nine selected samples (from six stations) of publicly available Tara Oceans Consortium data were used for the analysis.

### 16S rRNA amplicon analysis

A total of 17,604,113 (1,956,012.56 ± 645,294.51, mean ± sd) paired-end reads from nine water samples of Andaman region and 172,074,584 paired-end reads from Cape-Comorin water sample were generated. The reads were filtered to remove ambiguous bases using AmbiguityFiltering.pl of the NGS-QC toolkit^30^, no ambiguous bases were allowed, and reads shorter than 60 bp were discarded (-c 0, -n 60). Reads with 70% bases above Q20 were filtered using IlluQC_PRLL.pl. Primers were removed using cutadapt 1.18^31^, and remaining single-end reads post-filtration were eliminated using the repair.sh function of the bbmap toolkit, later the paired reads were assembled using FLASH (Fast Length Adjustment of SHort reads)^32^. Merged paired-end reads were imported in QIIME-2 for further analysis^33^. The reads were denoised and filtered for chimeric reads using dada2^34^. Low abundant Amplicon Sequence Variants (ASVs/features) with a total frequency of 10 across samples were removed and the resulting feature table was used for further analysis. Taxonomic annotation of ASVs was carried out using the q2-feature-classifier plugin after training on V3 region using Greengenes 13_5 reference database^35^. After taxonomic assignment, the relative abundance of phylum, genus, and species in each sample was calculated. Observed features and Shannon diversity were calculated to estimate alpha diversity. Inter-sample distances were calculated using Jaccard, Bray–Curtis, and UniFrac distances.

### Metagenomic data analysis

A total of 73,165,072 (12,194,178.67 ± 3,638,208.02) paired-end reads were generated from six samples from Andaman region. In addition, 985,580,126 paired-end reads (109,508,903 ± 83,144,900.59) were retrieved from nine selected samples of Tara Oceans Consortium. Metagenomic reads were filtered to remove ambiguous bases using AmbiguityFiltering.pl of the NGS-QC toolkit allowing zero ambiguous bases and sequences shorter than 60 bases were discarded (-c 0 -n 60). The resulting reads were filtered to remove low-quality reads. Further, quality filtration of reads was carried out with a cut-off of ≥ Q25 (N 1 -l 70 -s 25). Assembly of metagenomic reads from each sample was carried out using SOAPdenovo^36^ at different k-mers (53 to 109) and selected the best-assembled contigs for each sample based on different statistical parameters (Supplementary Fig. 1).

### Taxonomic assignment of metagenomic reads

Taxonomic annotation of metagenomic reads was carried out using Kaiju and Kraken2^37,38^. Kaiju classified 692,372,913 of 985,093,743 (70.28%) reads using default parameters. Kraken2 classified 340,218,793 of 985,093,743 (34.54%) reads. Species-level sequence abundance was inferred using Bracken^39^. Microbial genera present in all samples of coastal and open ocean groups with > 1% relative abundance were considered for defining core-microbiome composition.

### Reconstruction of ocean microbiome gene catalog

Open reading frames (ORFs) were predicted from contigs (contig size > 300 bp) of each sample separately using MetaGeneMark version 3.38^40^. The Ocean Microbial Reference Gene Catalog (OMRGC) consisting of 40,154,822 genes (from 243 ocean samples) constructed by the Tara Oceans Consortium was retrieved. For assessing the microbial gene diversity of the Indian Ocean region, we added 113,410 ORFs (> 100 bp nucleotide) predicted from Andaman data to OMRGC and carried out clustering of redundant set of genes using CD-HIT at 100% amino acid sequence identity and selected the longest gene from each cluster as a representative in updated non-redundant gene catalog^41^. The final non-redundant gene catalog consisted of 39,732,487 genes. Since differences in sequencing depth can create biases in gene quantification and comparative analysis, we subsampled 10 million reads (estimated based on Andaman data rarefaction) from Tara Oceans Consortium data and used those for further analysis.

### Gene quantification in Andaman and Tara Oceans samples and functional analysis

High-quality reads from Andaman and Tara Oceans Consortium samples (after subsampling) were mapped to the updated ocean microbiome gene catalog using soap2 of SOAPaligner version 2.21 with an identity cutoff of ≥ 90%^42^. Alignments of two types were considered for sequence-based profiling: (i) Entire paired-end read mapped to gene, and (ii) One end of paired-end reads mapped and the other read remained unmapped. In both cases, the read was counted as one copy. Further, the read count was normalized based on the length of the gene as b_i =_ x_i/_L_i._ The relative abundance of a gene within the sample was calculated as follows:

$${\text{a}}_{{\text{i}}} = {\text{b}}_{{\text{i}}} /\sum_{{\text{j}}} {\text{b}}_{{\text{j}}} = {\text{x}}_{{\text{i}}} {\text{/L}}_{{\text{i}}} /\sum_{{\text{j}}} {\text{x}}_{{\text{j}}} {\text{/L}}_{{\text{j}}}$$

where *a*_*i*_ is the relative abundance of gene in sample *S*, *x*_*i*_ is the number of time gene *i* was detected in sample *S* (i.e. the number of mapped reads), *L*_*i*_ is the length of gene *i*, *j* is all the genes (*j* number of genes) and *b*_*i*_ is copy number of gene *i* in sequence data from sample *S*^43,44^.

A total of 5,493,722 genes were present in at least one out of 15 samples. We filtered the quantified genes based on the two criteria: (i) genes should be present in more than 20% of the samples (3 out of 15 samples), and (ii) genes with > 0.05% relative abundance was reincorporated to avoid losing highly abundant genes that might be excluded in the previous criteria. After applying these gene exclusion criteria, 1,867,938 genes were selected and used for further analysis. Functional annotation of metagenomic reads and genes is needed to gain functional insights^45–47^ and was performed for 5,493,722 genes using eggNOG-mapper v2 with eggnog 5.0 database using diamond at default parameters^48,49^. The cumulative abundance of each KO and CAZy gene family was also calculated.

### Identification of antibiotic resistance genes

Antibiotic resistance genes were identified using Resistance Gene Identifier (RGI)^50^. From the three different prediction criteria provided by RGI i.e., perfect, strict, and loose options based on different types of hits in homology search, we used the “strict” criteria for the detection of ARGs in the updated ocean microbiome gene catalog. We also calculated the cumulative abundance of each Antibiotic Resistance Ontology (ARO), AMR gene families, drug classes, and antibiotic resistance mechanisms of ARGs detected in coastal water and open ocean samples.

### Analyses based on *Synechococcus* and *Prochlorococcus* genomes

We retrieved 1100 *Prochlorococcus* genomes and 405 *Synechococcus* genomes from the NCBI GenBank database (including complete and draft genomes). Custom databases were constructed for both genera separately using the above-mentioned genomes. The databases were indexed using bowtie2 and the abundance of each genome in samples was quantified by mapping high-quality metagenomic reads from Andaman and Tara Oceans Consortium samples against the custom genome database using bowtie-2. We proceeded with analyzing the top 100 abundant genomes from both coastal and open ocean samples. *Synechococcus* represented 85 out of 100 top abundant genomes in coastal samples, while 81 out of 100 top abundant genomes in open ocean samples were from the *Prochlorococcus* genus. Open reading frames were predicted from all 166 genomes (81 + 85) using Prodigal. We constructed a non-redundant gene set for both *Prochlorococcus* and *Synechococcus* separately by clustering the genes using CD-HIT at 100% amino acid identity^41,51^. Functional annotation of both gene sets was carried out using eggNOG-mapper v2^48^. We also examined the orthologous genes present in both gene sets using OrthoFinder and OrthoVenn2^52,53^. We also predicted 16S rRNA gene sequences from these highly abundant *Synechococcus* (n = 85) and *Prochlorococcus* (n = 81) genomes using barrnap (https://github.com/tseemann/barrnap) to evaluate the phylogenetic relatedness/structure of highly abundant genomes in coastal and open ocean waters^54^. Only 45 and 70 16S rRNA gene sequences could be predicted from genomes of *Synechococcus* and *Prochlorococcus* respectively. The complete 16S rRNA gene sequences were aligned using the Clustal-W algorithm implemented in MEGA-X^55^. We also constructed a phylogenetic tree using the neighbor-joining method by introducing *E. coli* 16S rRNA gene as an outgroup.

### Statistical analysis

**Figure 1:**
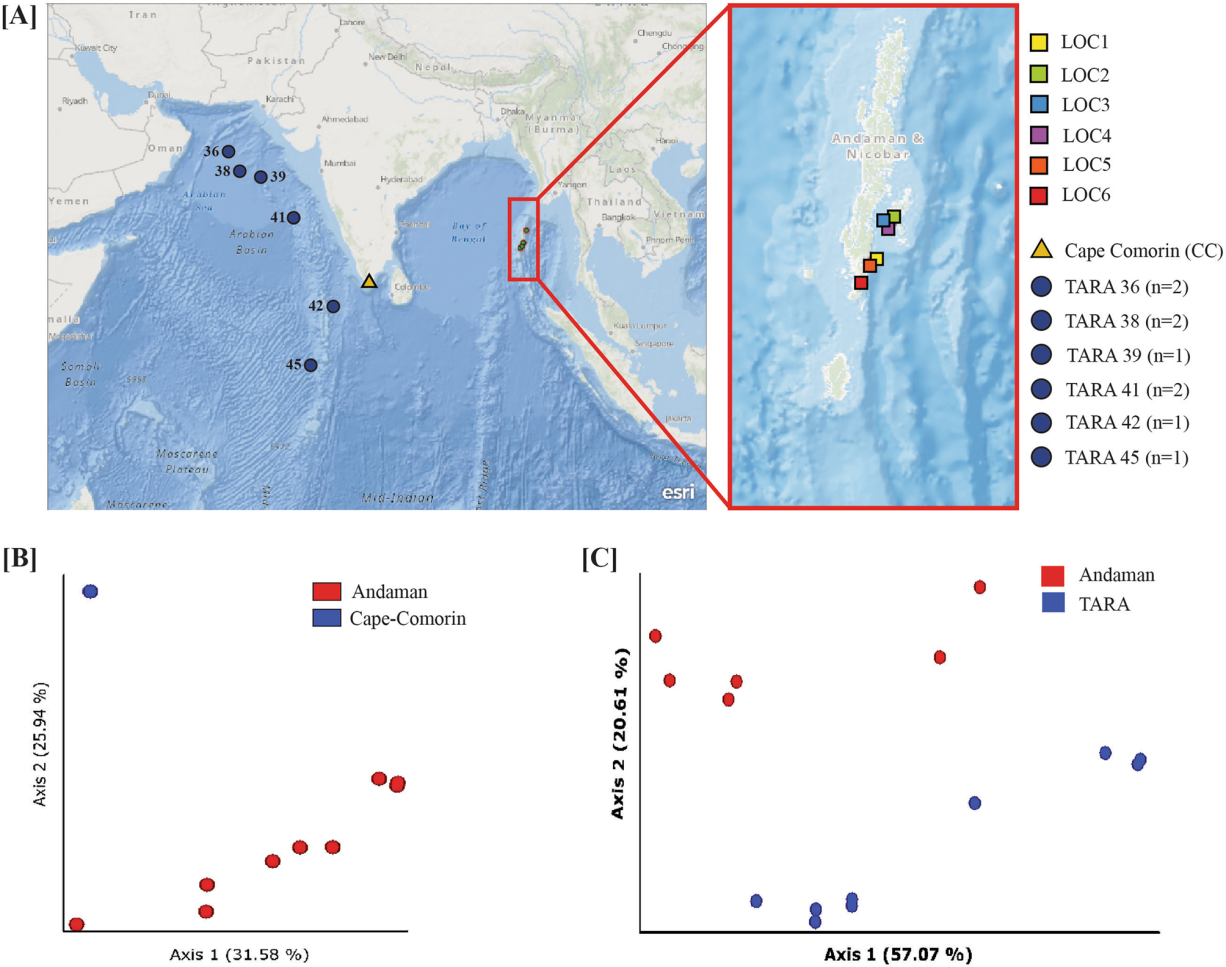
(**A**) The map shows locations (in colored rectangles) of coastal water sample collection sites from Andaman and Nicobar Islands. Blue colored circles represent Tara Oceans sampling stations in the Indian Ocean region for comparative analysis. (**B**) Principal coordinates analysis based on Bray–Curtis distance between 16S rRNA amplicon samples. Sample from Cape-Comorin clusters away from Andaman samples. (**C**) Principal coordinates analysis based on Bray–Curtis distance using microbial species abundance in each sample revealed a separation between Tara Oceans and Andaman samples; PCo-1 explained 57.07% of the variation.

Alpha and beta diversity of coastal and open oceans samples based on the taxonomic and functional composition of microbiomes by 16S rRNA amplicon and metagenome data were carried out using QIIME-2. Principal Coordinate Analysis (PCoA) was carried out using QIIME-2^33^. Differentially abundant species/genera were identified using LEfSe and Boruta^56,57^. The maps used in Fig. 1 were generated using ArcGIS Online (Esri). Plots were made using ggplot2 package in R^58^.

## Results

We collected surface water samples from six different coastal locations of the Andaman and Nicobar Islands and employed whole metagenomic and 16S rRNA gene amplicon-based approaches to study the microbiome composition of coastal water. Nine open ocean surface water samples of the Indian Ocean region from the Tara Oceans Consortium were selected for the comparative analysis (Supplementary Table 1, see methods for detailed information on sample collection). We generated whole metagenomic data from six samples of Andaman comprising 73,165,072 (12,194,178.67 ± 3,638,208.02) paired-end reads. A total of 985,580,126 (Average 109,508,903 ± 83,144,900.59) paired-end reads were retrieved for surface samples of the Indian Ocean region from Tara Oceans Consortium for comparative analysis (Supplementary Tables 1, 2, Fig. 1A, See Methods section).

We carried out 16S rRNA sequencing and analysis of ten samples including nine samples collected from coastal regions in Andaman and one sample from Cape-Comorin (CC). A total of 17,604,113 (1,956,012.56 ± 645,294.51) and 172,074,584 paired-end reads were generated for Andaman and CC respectively. Since the sequencing data from CC has significantly higher coverage, we extracted 5,562,311 reads (randomly) from the sample as a representative to avoid any bias in the analysis (Supplementary Table 3, Fig. 1A). After denoising and chimera removal using QIIME-2, 17,105,642 reads (90.77%) remained for further analysis. The analysis identified 23,303 ASVs present across all ten samples. Low-abundant ASVs were excluded and the remaining 19,604 features (ASVs) were used for calculating diversity matrices and examining taxonomic composition (See “Methods” section).

### Microbial richness and diversity of coastal water samples

Diversity and richness of Andaman and CC samples were evaluated using Shannon evenness, number of observed features (richness), Pielou’s evenness, and phylogenetic diversity (Faith’s PD) index. Higher microbial diversity (using Shannon evenness, number of observed features, and Pielou’s evenness) was observed at LOC3. The phylogenetic diversity (Faith’s PD) index showed the highest alpha diversity at CC. A variation of alpha diversity was observed in different sampling depths of LOC2 sites with LOC2-4 having the highest diversity (Supplementary Table 4, Supplementary Fig. 2A).

PCoA based on inter-sample distances (Bray–Curtis, Jaccard, and UniFrac) derived from 16S data showed close clustering of LOC2-1, LOC2-2, and LOC2-3, reasonably due to their geographical proximity. Andaman samples clustered far apart from the CC sample (Fig. 1B). PCoA based on inter-sample distances calculated using species abundance from metagenomic data resulted in distinct clustering of coastal and open ocean samples (Fig. 1C). The first and second principal coordinates explained 57.07 and 20.61% variations, respectively. These observations indicate the impact of geographical locations on overall microbiome composition.

### Abundance of *Synechococcus* species in coastal waters of Andaman Islands

**Figure 2:**
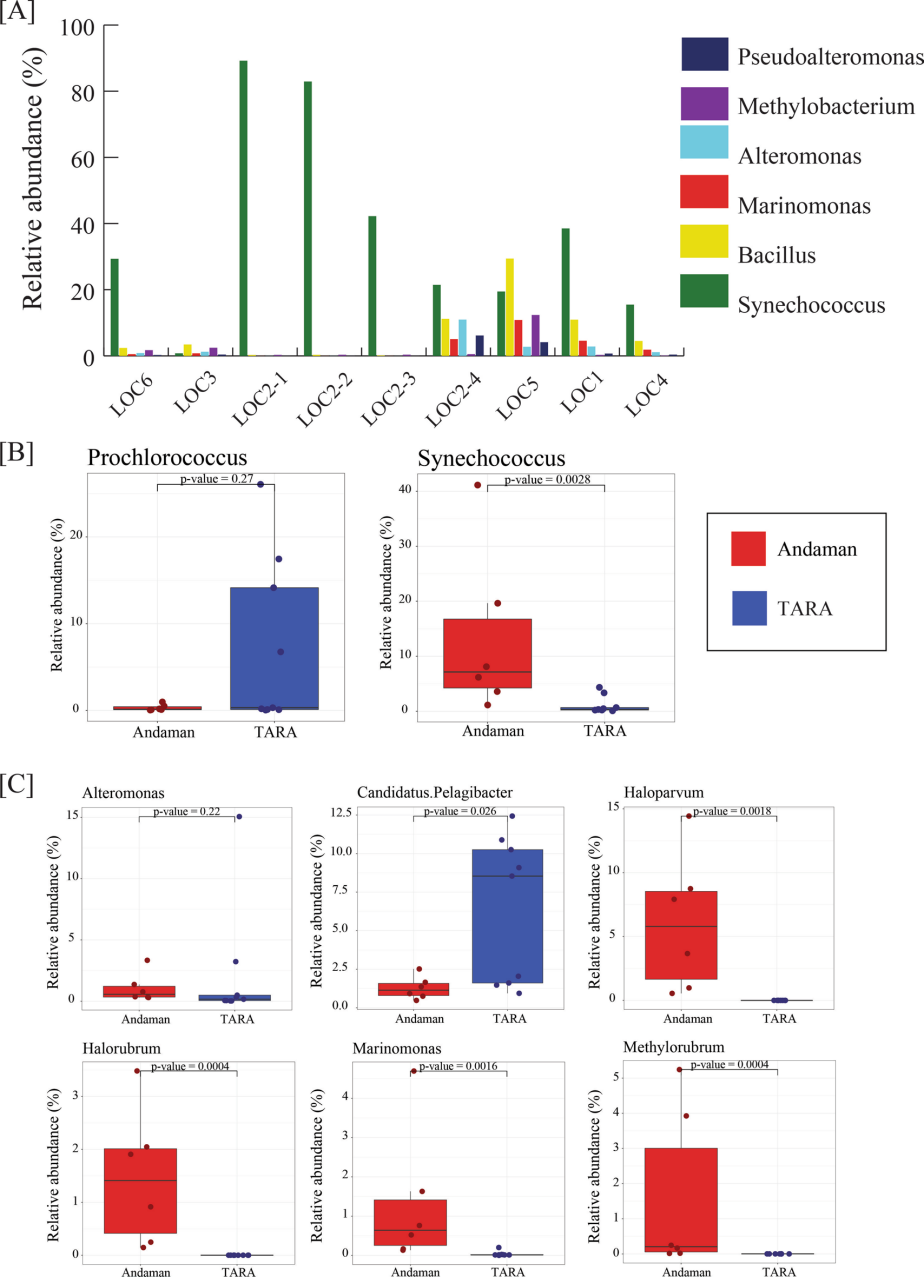
(**A**) Relative abundance of most abundant genera in 16S rRNA amplicon samples from coastal waters. (**B**) Figure shows the relative abundance of *Synechococcus* and *Prochlorococcus* in metagenomic data of open oceans (Tara Oceans) and coastal water (Andaman) samples. (**C**) Figure shows relative abundance of core genera (> 1% average abundance) identified in coastal (Andaman) and open ocean (Tara Oceans) samples. The relative abundance of all core genera (except *Alteromonas*) was significantly different in coastal and open oceans.

Taxonomic annotation of ASVs resulted in the identification of 59 phyla, 321 families, and 574 genera. Proteobacteria and Cyanobacteria (37% and 35% average relative abundance) showed the highest abundance followed by *Firmicutes*, *Actinobacteria*, *Bacteroidetes*, *Acidobacteria*, and *Gemmatimonadetes* in all samples including Andaman and CC. All these phyla showed > 1% abundance in all samples. At the family level, *Synechococcaceae*, *Bacillaceae*, and *Rhodobacteriaceae* (relative abundance 38.19, 11.97, and 5.37%) were the most abundant families followed by *Pelagibacteriaceae, Oceanospirallaceae*, *Alteromonadaceae*, *Methylobacteriaceae, Propionibacteriaceae,* and *Nocardioidaceae. Synechococcus* showed the highest abundance in Andaman samples with an average relative abundance of 37%. A core microbiome analysis was performed considering genera present in all samples with > 1% average relative abundance. *Synechococcus*, *Bacillus*, *Marinomonas*, *Alteromonas*, *Methylobacterium,* and *Pseudoalteromonas* were identified as core genera in Andaman samples. *Lutimonas* was found to be the most abundant genus in the CC sample, followed by *Anaerospora*, *Tenacibaculum*, *Thalassobius*, *Acidaminobacter*, *Nisaea*, *Propionibacterium,* and *Corynebacterium* (Fig. 2A, Supplementary Fig. 3A, B, C).

Further, we validated the genus-level microbial composition by taxonomic annotation of metagenomic reads using Kaiju (Supplementary Fig. 4A). *Synechococcus* (13.3%), *Haloparvum*, *Methylorubrum*, *Halorubrum*, *Candidatus*, *Marinomonas,* and *Alteromonas* were identified as core bacterial genera in Andaman samples. Whereas, *Prochlorococcus* (7.25%), *Candidatus Pelagibacter*, *Alteromonas,* and *Synechococcus* were detected as core bacterial genera in open ocean samples of Tara Oceans Consortium (Fig. 2B, C, Supplementary Table 5). The bacterial species abundance in coastal and open ocean samples analyzed using Kraken2 revealed *Methylorubrum populi*, *Synechococcus* sp*.* LTW-R, *Synechococcus* sp. TAK9802, *Synechococcus* sp. M16.1 and *Alteromonas macleodii* as the most abundant species with their average relative abundance ranging between 3.9 and 4.8% in coastal samples from Andaman. Whereas, *Prochlorococcus marinus,* showed the highest abundance (average relative abundance 12.5%) in open ocean samples (Supplementary Table 5).

**Figure 3:**
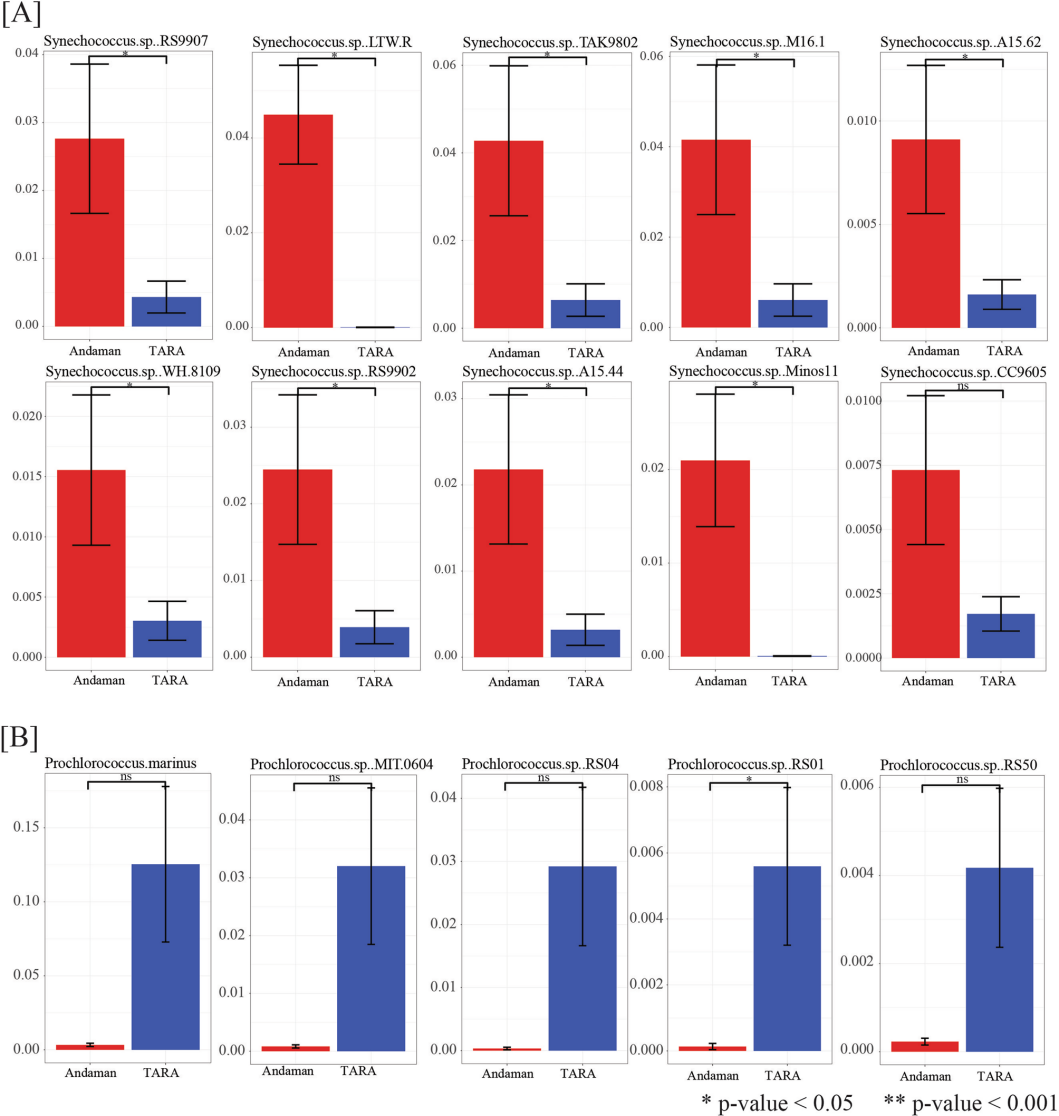
(**A**) 10 *Synechococcus* and (**B**) five *Prochlorococcus* species detected in most abundant 20 species in metagenomic data of coastal (Andaman) and open ocean (Tara Oceans) samples.

Analysis of the top 20 microbial species in coastal and open ocean samples indicated a higher number of species (10 out of 20) from *Synechococcus* in coastal samples (Fig. 3A, Supplementary Fig. 4B, Supplementary Table 5). On the other hand, the top 20 microbial species in open ocean samples were assigned to *Prochlorococcus* (five sp.), *Candidatus Pelagibacter* (six sp.), *Alteromonas* (four sp.), *Synechococcus* (four sp.), and *Pseudomonas resinovorans* (Fig. 3B, Supplementary Fig. 4C, Supplementary Table 5). Briefly, a higher abundance and richness of *Synechococcus* species in coastal water samples and a higher abundance of *Prochlorococcus* species were observed in open ocean samples.

### Differentially abundant microbial genera and species in coastal water and open ocean surface water samples

We detected 51 microbial genera exhibiting differential abundance between coastal water and open ocean surface water using LEfSe and Boruta. Out of 51 genera, 30 were Archaebacterial halophiles from class *Halobacteria*. These genera were highly abundant in coastal water samples. Many of these genera have been isolated from hypersaline environments, marine sediments, and soil (Supplementary Table 6). The *Hyphomonas* (class Alphaproteobacteria) was the only genus among the differentially abundant genera that exhibited higher abundance in the open ocean samples. This bacterium is a dimorphic, prosthecate bacteria ubiquitous in the marine environment^59^ (Supplementary Fig. 5, Supplementary Table 6).

Analysis using Boruta and LEfSe identified 47 differentially abundant microbial species (from kraken2 analysis) in coastal water and open ocean surface water. Out of these, 41 species showed significant abundance in coastal waters, and notably, 32 of these (~ 69.56%) were identified as salt-tolerant species (Supplementary Fig. 6, Supplementary Table 6). Five viruses of *Synechococcus* and *Prochlorococcus* (Supplementary Table 6) were found to be differentially abundant in open oceans based on the abundance values obtained using kraken2. Marine viruses have active roles in shaping the physiology and diversity of picocyanobacterial species (Supplementary Fig. 6)^60^.

### Higher diversity of *Synechococcus* genomes in coastal waters compared to *Prochlorococcus* in open ocean water samples

**Figure 4:**
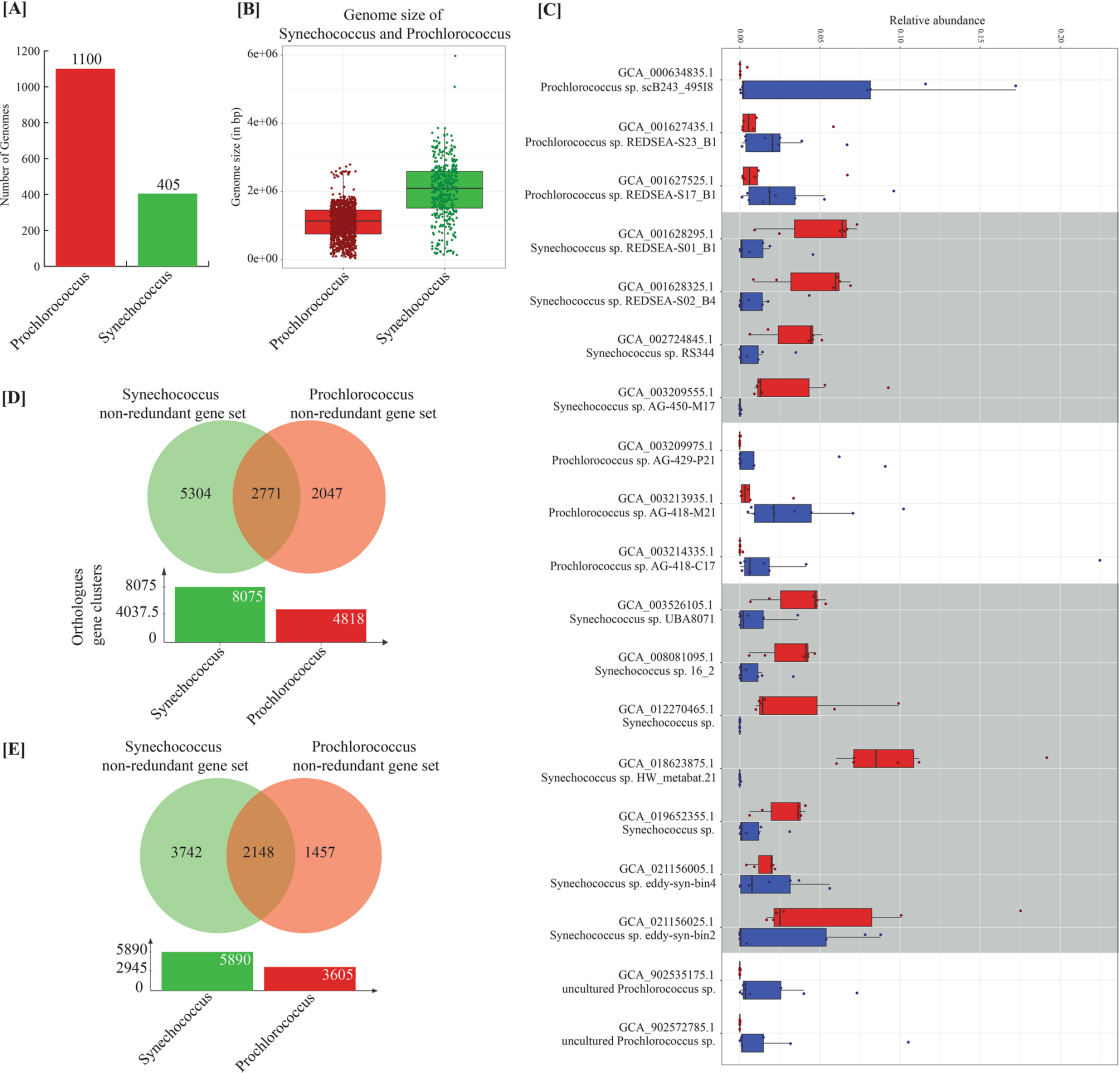
(**A**) Number of *Synechococcus* and *Prochlorococcus* genomes retrieved from NCBI Genbank database for analysis. (**B**) Box-plot representing genome size of *Synechococcus* and *Prochlorococcus* genomes used in the analysis. (**C**) Top abundant *Synechococcus* and *Prochlorococcus* genomes in Andaman and Tara Oceans samples based on alignment of metagenomic reads to genomes. Genomes highlighted in Grey were abundant in Andaman coastal samples. (**D**) Venn-diagram of orthologous gene clusters of *Synechococcus* and *Prochlorococcus*. The gene sets of *Synechococcus* and *Prochlorococcus* used for this analysis were constructed by clustering of ORFs predicted from both genera at 100% amino acid identity. (**E**) Venn-diagram of orthologous gene clusters of *Synechococcus* and *Prochlorococcus*. The gene sets of *Synechococcus* and *Prochlorococcus* used for this analysis were constructed by clustering of ORFs predicted from both genera at 90% amino acid identity.

To further explore the genome-level composition of the most abundant genera in coastal and open ocean samples, we constructed genome databases for both *Prochlorococcus* and *Synechococcus* by retrieving 1100 and 405 genomes, respectively from NCBI GenBank database (Fig. 4A, B). The average genome size of *Prochlorococcus* is smaller than *Synechococcus* (*p* value = 1.750e−88) (Fig. 4B). Whereas, the variation in genome size of *Synechococcus* is significantly higher than *Prochlorococcus*. We calculated the abundance of all 1505 genomes in all 15 samples and used the top 100 genomes in both datasets for further analysis. In the resulting data, 81 out of the top 100 genomes in the open ocean samples were from the *Prochlorococcus* genus. Similarly, 85 out of the top 100 genomes in coastal water samples were from the *Synechococcus* genus (Supplementary Table 7). Among the top 20 genomes in the data, 11 *Synechococcus* genomes were abundant in coastal samples (Fig. 4C, Supplementary Table 7) and others were abundant in the open ocean group.

We also constructed a phylogenetic tree using 16S rRNA sequences predicted from the most abundant *Synechococcus* (n = 45 out of 85) and *Prochlorococcus* (n = 70 out of 81) genomes in coastal and open ocean microbiomes. Higher phylogenetic diversity was observed in a phylogenetic tree constructed using *Synechococcus* genomes, whereas the most abundant *Prochlorococcus* genomes from open ocean samples formed only two major clades (Supplementary Fig. 7).

### Non-redundant gene set of most abundant *Synechococcus* and *Prochlorococcus* genomes and their functional insights

To evaluate the functional potential of top abundant *Synechococcus* (n = 85) and *Prochlorococcus* (n = 81) genomes in coastal and open ocean waters microbiome, we constructed non-redundant gene sets for each genus by predicting genes from the top abundant genomes (see methods). *Synechococcus* and *Prochlorococcus* gene sets contained 137,371 and 88,718 non-redundant genes respectively. The difference in the number of genes in both datasets reflects the differences in the respective genome sizes of the two cyanobacteria.

We also analyzed the highly abundant functions (in terms of KOs) in *Prochlorococcus* and *Synechococcus* gene sets. Highly enriched KOs in both genera were involved in photoinduced damage repair and oxidative stress (*ftsH*, *phrB*, *gst*), ABC type transporter proteins, chlorophyll biosynthesis-related protein (K00218), carbohydrate metabolism (galE, *rfbD*), amino acid metabolism (K01802) and transcription (Supplementary Table 8). Differentially represented functions (KOs) in both gene sets were evaluated by the ratio of the number of genes assigned to each KO in *Synechococcus* to the number of genes assigned to each KO in *Prochlorococcus* and vice versa (Syn/Pro and Pro/Syn ratio) (Supplementary Table 8).

### Identification and annotation of orthologous gene clusters in non-redundant gene sets of the most abundant *Synechococcus* and *Prochlorococcus* genomes

OrthoFinder and OrthoVenn2 were used to examine the orthogroups present in *Synechococcus* and *Prochlorococcus* gene sets. Analysis using OrthoVenn2 at 100% identity cut-off resulted in 8075 orthologous gene clusters from *Synechococcus* and 4,818 orthologous gene clusters from *Prochlorococcus*. Of these, 5304 and 2047 unique orthologous gene clusters were identified in *Synechococcus* and *Prochlorococcus,* respectively (Fig. 4D). Further validation using gene sets constructed at a 90% identity threshold identified 5890 and 3605 orthologous genes (total of 7347) clusters in *Synechococcus* and *Prochlorococcus,* respectively (Fig. 4E). At the 90% identity threshold, 3742 and 1457 unique orthologous gene clusters were identified in *Synechococcus* and *Prochlorococcus,* respectively. The longest genes from each cluster were selected as representative and annotated based on a homology search against the nr database using DIAMOND. Out of 7347 genes, 4307 (58.62%) representative genes were annotated using the criteria of ≥ 90% identity and ≥ 80% query coverage (Supplementary Tables 9, 10).

Confirmatory analysis using OrthoFinder revealed 9295 orthogroups (containing at least two genes per cluster) within *Synechococcus* (7263 orthogroups) and *Prochlorococcus* (4007 orthogroups). Of these, only a fraction, 64.20% (5968 out of 9295) were annotated using the nr database. In particular, we identified 5288 unique orthogroups in *Synechococcus* and 2032 in *Prochlorococcus*. A higher number of unique gene clusters present in *Synechococcus* genera indicates the potential of *Synechococcus* genomes to carry out diverse and distinct functions compared to *Prochlorococcus* genomes (Supplementary Tables 9, 10). A large part of these clusters was annotated as hypothetical proteins using DIAMOND-BLAST against the nr database.

We also examined the presence of photosynthesis-related proteins in both *Synechococcus* and *Prochlorococcus* orthologous gene sets using the KEGG database. Photosynthesis-related proteins are represented as three major groups in the KEGG database namely, photosystem and electron transport system, antenna proteins, and anoxygenic photosystem. Among the orthologous gene sets identified via OrthoFinder, we found 28 KOs associated with the photosystem and electron transport system within *Synechococcus*. In contrast, *Prochlorococcus* exhibited only 11 KOs related to these functions. Similarly, 18 KOs in orthologous gene sets (OrthoFinder) of *Synechococcus* and 2 KOs in orthologous gene sets (OrthoFinder) of *Prochlorococcus,* were mapped to antenna proteins.

### Updated Ocean microbiome gene catalog

Sample wise assembly and gene prediction from Andaman samples resulted in 113,410 non redundant genes of > 100 bp nucleotides length (Supplementary Table 11). We updated the ocean microbiome gene catalog by adding 113,410 genes to the Ocean Microbial Reference Gene Catalog (OMRGC). The updated non-redundant catalog contained 39,732,487 genes. A reduction in the number of genes in the updated gene catalog indicates the redundancy in OMRGC. The updated gene catalog contains 93,863 genes from the Andaman region. Out of these, 93,172 (99.26%) genes were part of unique clusters and are not covered in OMRGC. Further analysis of 691 genes that were clustered with genes from OMRGC resulted in the identification of 298 representative genes from Andaman that replaced OMRGC genes with more than two-fold their length. Only a limited number of newly added genes could be annotated using eggNOG-mapper v2. Only 53,934 (57.46%) genes out of 93,863 genes were annotated using DIAMOND against the nr database with ≥ 90% identity and ≥ 80% query coverage (Supplementary Table s 12). Further, only 2688 genes (2.86%) were assigned molecular functions in KEGG, 45 (0.048%) were assigned by CAZy, 1608 (1.71%) were assigned to EC classes, and 3919 (4.17%) to COG classes. Thus, a total of 53,934 (57.46%) genes out of 93,863 genes were successfully annotated with a specific function. The presence of a large number of unannotated proteins might represent new and previously unknown functional diversity from coastal surface waters. We looked for the KOs involved in photosynthesis and found 26 genes annotated as photosynthetic proteins in genes newly contributed from our study. Similarly, 12 genes were involved in the betaine biosynthesis pathway, which is a compatible solute and provides the cells with adaptations of osmotic stress.

### Functional role of ocean microbiome in the coastal and open ocean region

**Figure 5:**
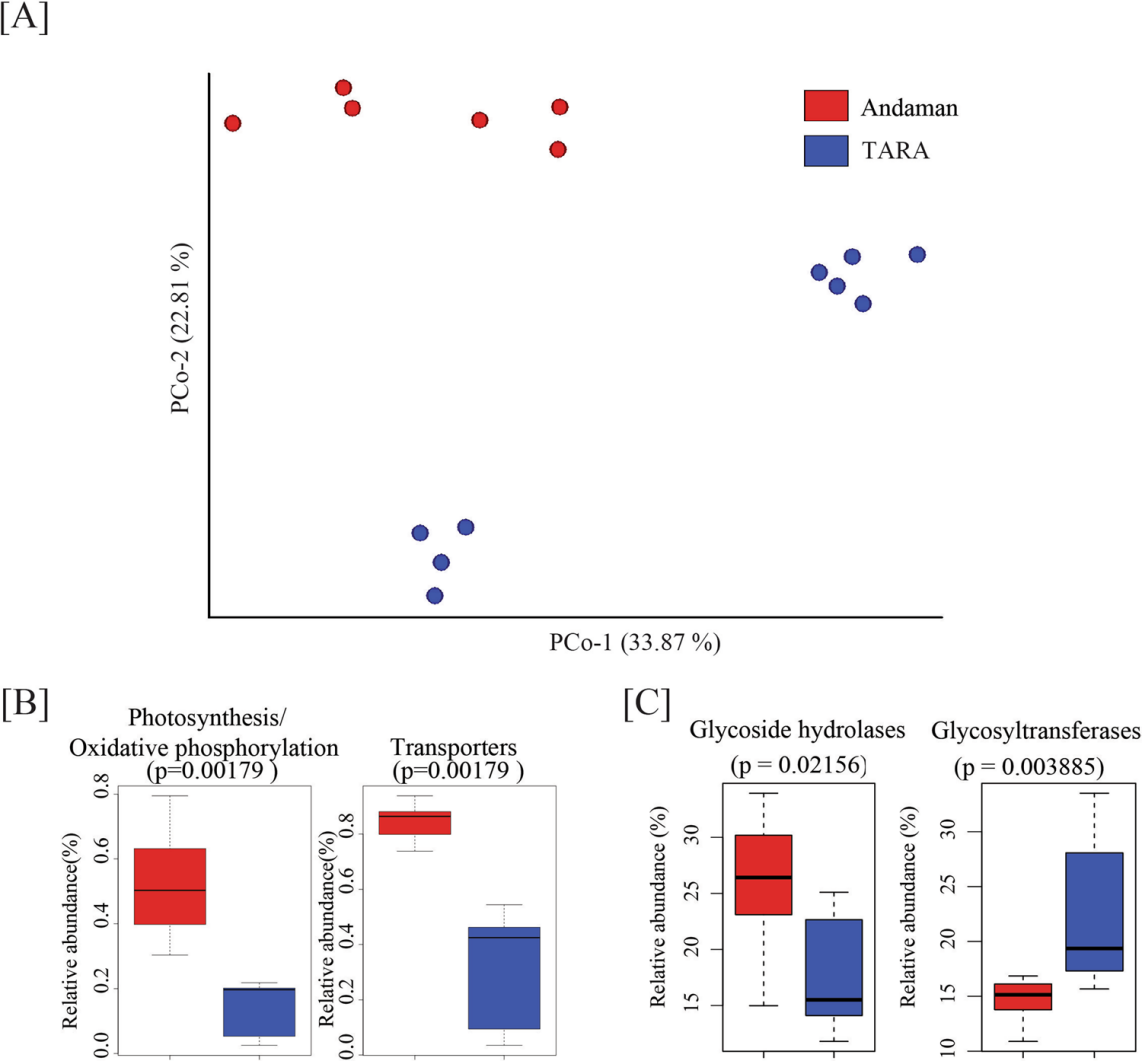
(**A**) Principal coordinates analysis based on relative abundance of KOs of coastal and open ocean samples. (**B**) Relative abundance of photosynthesis/oxidative phosphorylation related functions and transporters in Andaman and Tara Oceans samples. (**C**) Relative abundance of Glycoside Hydrolase and Glycosyltransferase CAZy gene families in Andaman and Tara Oceans samples.

We calculated the abundance of genes from the updated ocean microbiome gene catalog in all coastal and open ocean samples. Out of a total of 39,732,487 genes, 5,493,722 (13.82%) were identified in at least one surface water sample. A total of 2,250,966 (40.97%) genes were assigned with KEGG Orthologs (KO) using the KEGG database. Functional analysis was conducted on 1,867,938 genes detected in over 20% of the samples (i.e., at least 3 out of 15 samples) and exhibiting a relative abundance exceeding 0.05% per sample. Alpha diversity based on Shannon evenness and observed KOs in coastal and open ocean samples were also calculated (Supplementary Table 13). Beta diversity analysis using Jaccard distance indicated distinct clustering of coastal and open ocean samples (Fig. 5A).

Functions related to the transport of various substrates (7 transporters out of top 20 abundant functions), which include transporters for the acquisition of iron and copper (*ABC.CD.P*, *TC.FEV.OM*), amino acid transporters (*livK*), genes for transport of ammonium (*amt*) and transporters for the acquisition of sugars and branched amino acids were found in coastal water samples. Genes involved in DNA replication and repair (*rpoC*, *phrB*), amino acid metabolism (*glnA*, *gcvT*, *DMGDH*), photosynthesis (*psbA*), and photoreactivation after UV damage (*phrB*) also showed higher abundance in coastal samples. Genes involved in osmoregulation and oxidative stress (*DMGDH*, *GST*, sugar transporters, *ftsH*) were also abundant in coastal waters.

Open ocean samples showed a higher abundance of genes involved in DNA replication and repair (*polA*, *uvrD*, *MGME1*), purine nucleotide biosynthesis (*nrdA*, *nodU*), photosynthesis and electron transport (*petF*), heat shock proteins, and chaperone proteins (*groES*, *PEO1*, *groEL*, *ibpA*) and complex carbohydrate biosynthesis related functions (*UGDH*, *TSTA3*, *tagD*, *galE*). Iron acquisition transporter (*TC.FEV.OM*) was also among the most abundant functions in the open ocean microbiome.

Genes involved in photosynthesis and oxidative phosphorylation (*apcE, atpB, coxC, ctaE, cpcC, cpeA*) were differentially abundant in Andaman coastal water samples. Many transporters related to peptide transport (*ABC.PE.S* & *ABC.PE.P1*, *aapQ*), and polyamine uptake were also differentially abundant which serve as carbon, nitrogen, and/or energy source for marine bacterioplankton. Many other differentially abundant genes in coastal samples were transporters for sugar transport (fructose transport system, multiple sugar transport system ATP-binding protein) and Na^+^/H^+^ antiporter involved in pH and ionic homeostasis. Other differentially abundant genes related to amino acid biosynthesis pathways, stress response, purine nucleotide synthesis (*pncA*, *pncB*, *gpt*), metal stress (*chrA*, K07240), NAD biosynthesis, and carbohydrate metabolism genes, hydrocarbon degradation (*cntA*) and dehalogenases (*queG*) were also found at higher abundance in coastal samples than open ocean samples. Differentially abundant functions in open ocean samples include DNA replication and repair, carbohydrate biosynthesis (*galE*, glf, hddA), and iron homeostasis (*iscA*, *erpA*).

### Carbohydrate metabolizing genes analysis

A higher abundance of carbohydrate metabolizing genes in coastal and open ocean samples identified by KEGG analysis motivated an in-depth investigation of carbohydrate metabolizing enzyme families using the carbohydrate-active enzyme (CAZy) database. The updated gene catalog revealed a total of 58,207 CAZy genes. Differentially abundant CAZy genes in coastal and open ocean water were detected using Boruta and LEfSe. This analysis showed higher abundance of complex carbohydrate hydrolysis related functions like beta-NAGsidases, and glycoside hydrolases with varying substrate specificities in coastal waters. The abundance of genes involved in carbohydrate degradation and hydrolysis functions indicates more heterotrophic activity in coastal waters. In addition, a higher abundance of trehalose phosphate synthase (GT20) was also observed in coastal samples compared to open ocean water. Whereas open ocean samples showed a differential abundance of complex CAZy families transferases catalyzing various complex polysaccharide synthesis (GT25, GT31, GT15, GT47, etc.). Pectin degradation-related CAZy families (PL4, PL11) were also found to be abundant in open ocean samples (Fig. 5C).

### Antibiotic resistance genes analysis

**Figure 6:**
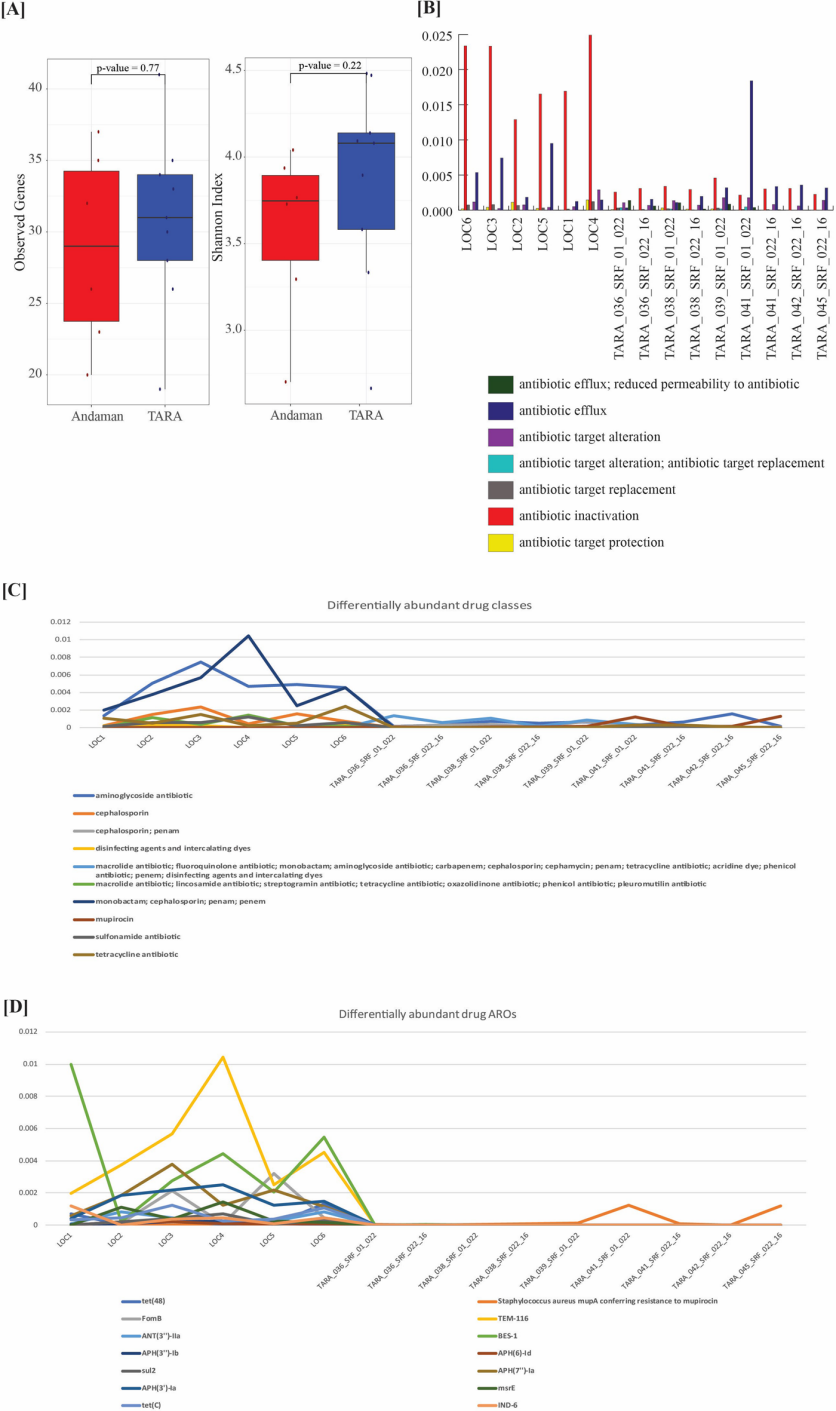
(**A**) Box plot based on alpha-diversity of Antibiotic Resistance Ontology (ARO) classified by RGI. The number of genes (observed AROs) and Shannon Index were calculated based on the proportion of differentially abundant AROs identified using LEfSe and Boruta. (**B**) Relative proportions of various Antibiotic resistance mechanisms detected in samples from coastal and open ocean samples. (**C**) Relative proportion of differentially abundant drug classes in which the resistance was detected in samples from coastal and open ocean waters. (**D**) Relative proportions of Antibiotic Resistance Ontology (ARO) in each sample. All AROs were highly abundant in coastal water samples compared to the open ocean.

To examine the impact of the higher human activity in coastal regions, we analyzed the composition of antibiotic resistance genes in coastal waters and compared it with open ocean samples. Antibiotic resistance genes were identified from 5,493,722 genes represented in the updated gene catalog using RGI. Differentially abundant Antibiotic Resistance Ontology (ARO), AMR gene families, and drug classes were identified using LEfSe and Boruta (Fig. 6). Genes categorized (by RGI) as conferring resistance to aminoglycoside, beta-lactam (monobactam; cephalosporin; penam; penem) and tetracycline antibiotics were identified as differentially abundant in coastal samples. Most of the differentially abundant AMR functions were in higher abundance in coastal waters compared to the open ocean. Analysis of AROs indicated a higher abundance of TEM-116, BES-1, APH(7″)-Ia, APH(3′)-Ia, FomB which correspond to beta-lactam, and aminoglycoside antibiotic resistance in coastal waters. The top five differentially abundant AMR gene families in Andaman coastal waters include TEM beta-lactamase, BES beta-lactamase, APH(7″), ADC beta-lactamases (with undetermined carbapenemase activity), and major facilitator superfamily (MFS) antibiotic efflux pump (Fig. 6, Supplementary Table 14).

## Discussion

The study of the microbial composition and interactions in specific ocean regions promises to enhance our understanding of the functional potential of ocean microbiomes and their roles in maintaining healthy marine ecosystems. While previous studies have focused on the open ocean, the microbial composition of coastal waters remains understudied. This study reveals the taxonomic and functional diversity of the coastal region of Andaman using the 16S rRNA gene amplicon and whole metagenomic data analysis. The comparative analysis of coastal water microbiome with samples from the open ocean region of marine pelagic biomes of ARAB and MONS retrieved from the Tara Oceans Consortium revealed key differences between the two ecosystems.

One of the main outcomes of this study is the construction of an updated ocean microbiome gene catalog by the inclusion of 93,172 unique genes from coastal samples that will help to unravel the yet unexplored functional diversity of the coastal waters in Andaman with potential biotechnological applications^24,61^. Notably, the *Synechococcus* species was significantly abundant in Andaman coastal water compared to open ocean samples from the Tara Oceans Consortium, consistent with previous reports of their widespread distribution in surface waters^62^. As a photosynthetic bacterium, *Synechococcus* exhibits efficient adaptations to variable salinity, light intensity, and temperature^63^. Certain strains of *Synechococcus* demonstrate distinct photosynthetic activity during the summer season, contrasting with other seasons^64^. On the contrary, *Prochlorococcus* genomes showed higher abundance (not significant) in open ocean samples compared to the coastal waters of Andaman. *Prochlorococcus* thrives in sunlit and well-oxygenated, nutrient-poor, tropical, and subtropical waters^62–64^.

*Synechococcus* and *Prochlorococcus* differ in their light-harvesting apparatus, with *Synechococcus* utilizing chlorophyll a and *Prochlorococcus* relying on divinyl chlorophylls a and b^65^. In-depth analysis constructing comprehensive genome databases of *Synechococcus* and *Prochlorococcus* revealed a higher abundance of genomes reconstructed from metagenomes (MAGs) in both coastal and open ocean waters indicating the yet under-explored diversity of *Synechococcus* and *Prochlorococcus* species. Also, *Synechococcus* genomes were significantly larger and more variable in size compared to *Prochlorococcus* genomes^65^.

Analysis of non-redundant gene sets (nr gene sets) of highly abundant genomes of *Synechococcus* and *Prochlorococcus* revealed the functional roles of these species in coastal and open ocean waters. We observed a higher abundance of *psbA* gene, which encodes D1 protein of PSII, in coastal water samples. This gene is known to be highly expressed in *Synechococcus sp. WH7803* under changing light conditions, indicating the adaptability of *Synechococcus* species to light stress in coastal waters^66^. The presence of terrestrial material, algae, and suspended biomass in coastal waters leads to different light quality compared to open oceans and may influence the distribution of *Synechococcus* and *Prochlorococcus* species. The incidence of fewer pigment-related genes in the *Prochlorococcus* gene set signifies its adaptation to relatively stable light conditions prevalent in open ocean environments. Conversely, *Synechococcus* genomes showcase a broader spectrum of pigment types, likely an adaptive strategy to cope with varying light regimes. The diverse array of pigments in *Synechococcus* genomes from coastal waters equips them to acclimate to fluctuating light conditions and nutrient-rich environments. In contrast, *Prochlorococcus* exhibits limited functional diversity and thrives primarily in relatively uniform oligotrophic waters.

Coastal waters exhibit variable salinity ranges and the presence of coastal lagoons and salt marshes can result in hypersaline conditions. Differential abundance of halophile genera (*Haloarcula* and *Haloquandratum*) and compatible solute biosynthesis-related KOs (e.g. KO10112) in coastal waters indicated higher adaptations like osmotic adjustments in coastal microbial community^67,68^. Several salt-tolerant, halophilic, or haloalkaliphilic bacterial/archaeal species were found to be differentially abundant in the coastal waters of Andaman. Various strains of differentially abundant species have been isolated from environments of high-salinity. (Supplementary Table 6). Specifically, the lack of water channel protein *AqpZ*, a channel that permits osmotically driven movement of water in both directions, and is involved in the osmoregulation and maintenance of cell turgor during volume expansion in rapidly growing cells, in almost all picoplanktonic cyanobacteria (with the exception of the coastal euryhaline strain *Synechococcus sp. strain WH5701*)^65^ further highlights the possibility of adaptations like osmotic adjustments in coastal microbial communities.

Due to their susceptibility to anthropogenic activities and associated stress conditions, coastal waters create an environment conducive to the presence and accumulation of ARGs^69,70^. In this study, the analysis of ARGs in coastal and open ocean water samples revealed the presence of 64 AMR gene families in the Andaman region, in which the genes conferring resistance to aminoglycoside, beta-lactam, and tetracycline antibiotics were highly abundant in coastal waters. We also observed NDM beta-lactamase in Andaman samples and five Indian Ocean samples (not significantly different). The NDM class (New Delhi metallo-beta-lactamase) of beta-lactamase enzymes hydrolyzes a wide range of beta-lactam antibiotics, including carbapenems. These antibiotics are critical for combating drug-resistant bacterial infections. Originally reported in India, the prevalence of NDM has expanded globally, posing a significant public health concern^23,71,72^. Most of the differentially abundant ARGs showed higher abundance in coastal waters compared to the open ocean. A recent study analyzing globally distributed Tara Oceans data identified tetracycline and beta-lactam resistance as prevalent ARGs^73^. Fosmidomycin, quinolone, and bacitracin ARGs were also reported to be higher in coastal biomes in Tara Oceans data. A recent study reported higher abundance of quinolone ARGs in coastal waters from China^69^. Additionally, in the Indian Ocean Commission (IOC) study encompassing various island countries within the Indian Ocean, carbapenemase-producing *Enterobacteriaceae* and extended-spectrum beta-lactamase were identified as principal concerns for human and animal health^74^.

In summary, this study revealed the microbiome composition of the coastal waters of Andaman Islands. Comparative analysis with selected open ocean samples from Tara Oceans consortium revealed differences between coastal and open ocean habitats. Increased abundance, prevalence of diverse species, and distinct functional composition of *Synechococcus* genus in coastal samples indicate their adaptability to variable environmental conditions and nutrient enrichment. A detailed analysis of abundant *Synechococcus* genomes revealed enrichment of photosynthesis pigment-related genes, suggesting their adaptation to the variable light conditions in coastal waters. The differential abundance of halophile species observed in coastal waters corroborates well with highly variable salinity in coastal areas. The inclusion of 93,172 unique genes from Andaman coastal water samples with a large number of unannotated genes in the updated gene catalog indicated the yet unexplored functional potential of these areas. This study also highlights the significance of monitoring coastal waters for antibiotic resistance due to human impacts and the potential risk of Horizontal Gene Transfer (HGT) of Antimicrobial resistance genes.

Even though this study was performed with a limited number of samples from a coastal region of a single geographical area, it represents the first metagenomic exploration of Indian coastal locations from the Andaman and Nicobar Islands. Considering the long coastline (~ 7500 km) of India with diverse environmental features, such as mangroves, coral reefs, seagrass beds, estuaries and lagoons, backwaters, etc., the knowledge and understanding of taxonomic and functional diversity in Indian coastal waters is much needed along with the studies to examine the impacts of anthropogenic activities on a much larger scale. Further studies based on longitudinal sampling with comprehensive environmental metadata from diverse habitats with different levels of anthropogenic impacts will provide more insights into microbial community variation due to high human activity. Exploring the microbial composition of coastal waters from diverse geographical regions will shed light on the underexplored diversity of *Synechococcus* genotypes.

### Supplementary Information

Supplementary Information 1.

Supplementary Information 2.

Supplementary Information 3.

Supplementary Information 4.

Supplementary Information 5.

Supplementary Information 6.

Supplementary Information 7.

Supplementary Information 8.

Supplementary Information 9.

Supplementary Information 10.

Supplementary Information 11.

Supplementary Information 12.

Supplementary Information 13.

Supplementary Information 14.

Supplementary Information 15.

## Data Availability

The raw sequencing data is available on NCBI BioProject database under study accession PRJNA822508 and PRJNA295549.
