# Supplementary material for: Metagenomic exploration of Andaman region of the Indian Ocean
Source: Sci Rep. 2024 Feb 1;14:2717. doi: 10.1038/s41598-024-53190-1 (PMC10834444; doi:10.1038/s41598-024-53190-1)
Supplement: Supplementary file 1 — Supplementary Information 1. [file 41598_2024_53190_MOESM1_ESM.pdf]

Additional information to manuscript titled: Metagenomic exploration of Andaman region of the Indian Ocean

Authors: Vishnu Prasoodanan PK[1#], Sudhir Kumar[1#], Darshan B Dhakan[1], Prashant Waiker[1], Rituja Saxena[1], Vineet K Sharma[1*]

Affiliation: [1]MetaBioSys Group, Department of Biological Sciences, Indian Institute of Science Education and Research Bhopal, Bhopal, India

[#]These authors contributed equally to the work

***Corresponding Author**: MetaBioSys Group, Department of Biological Sciences, Indian Institute of Science Education and Research Bhopal, Bhopal, India vineetks@iiserb.ac.in

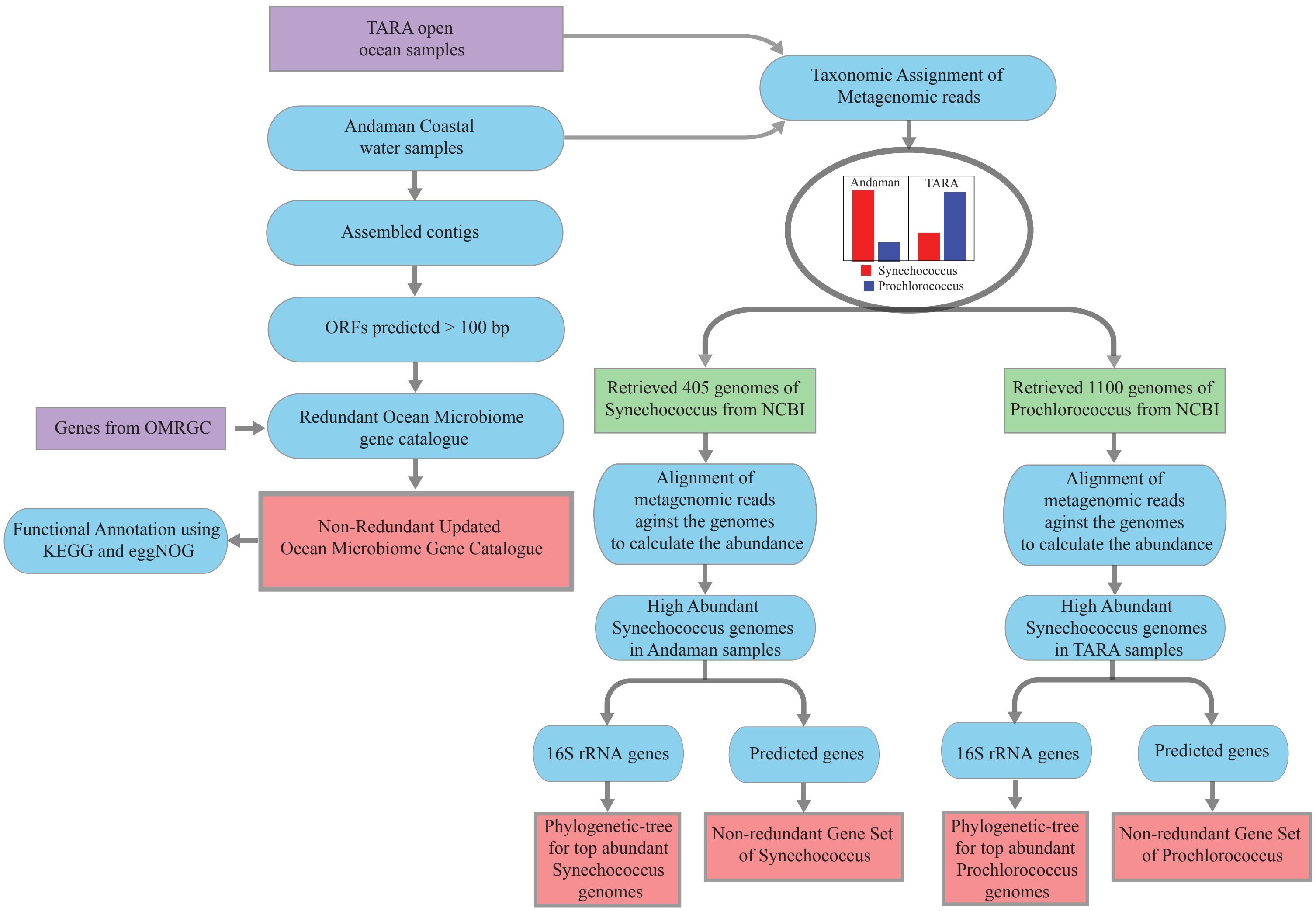

Supplementary Figure 1: Flow chart representing the analysis pipeline used to explore metagenomic data used in this study.

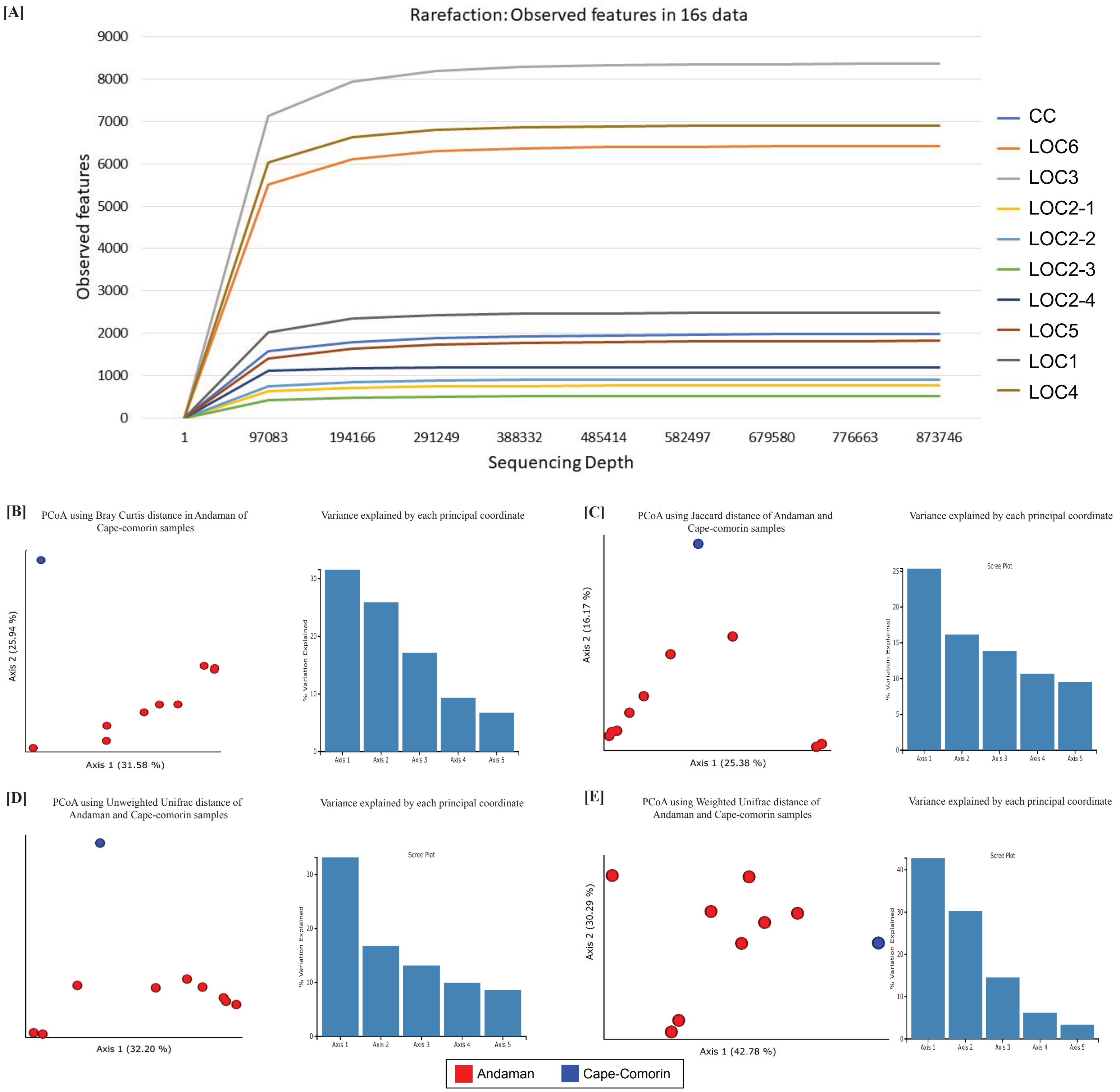

Supplementary Figure 2 [A] Rarefaction curve of observed features (ASVs) as a function of sampling depth for Andaman and Cape comorin coastal water samples. **[B–E]** Principal Coordinate analysis plots and their respective scree plots showing variance explained by each Principal Coordinate for Coastal Amplicon data. (**[B]** Bray Curtis distance; **[C]** Jaccard distance; **[D]** Unweighted UniFrac distance; **[E]** Weighted UniFrac distance.

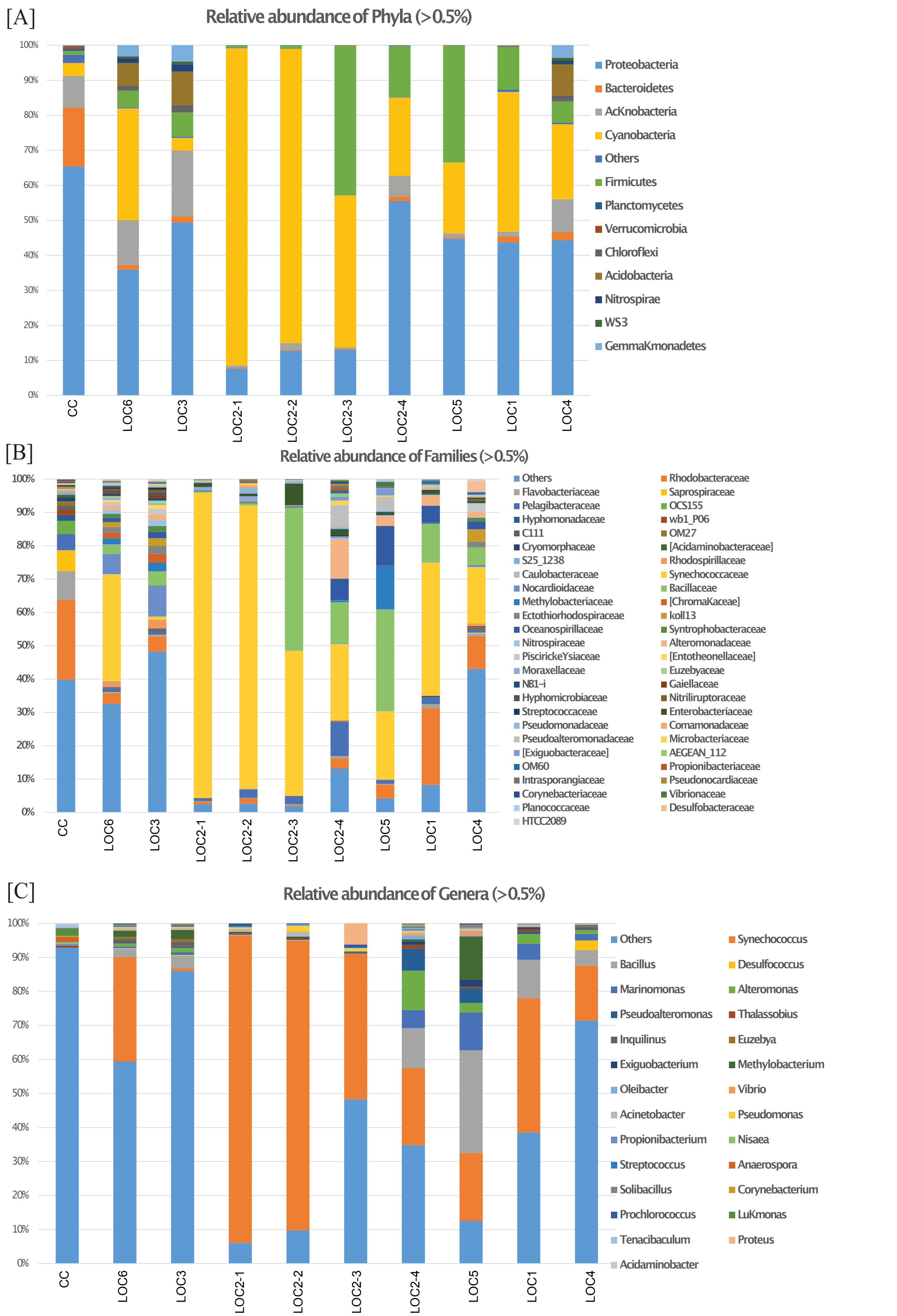

SupplementaryFigure3: Relative abundance (with criteria >0.5%) of **[A]** Phyla, **[B]** Family, and **[C]** Genera found in Coastal water samples using Amplicon data.

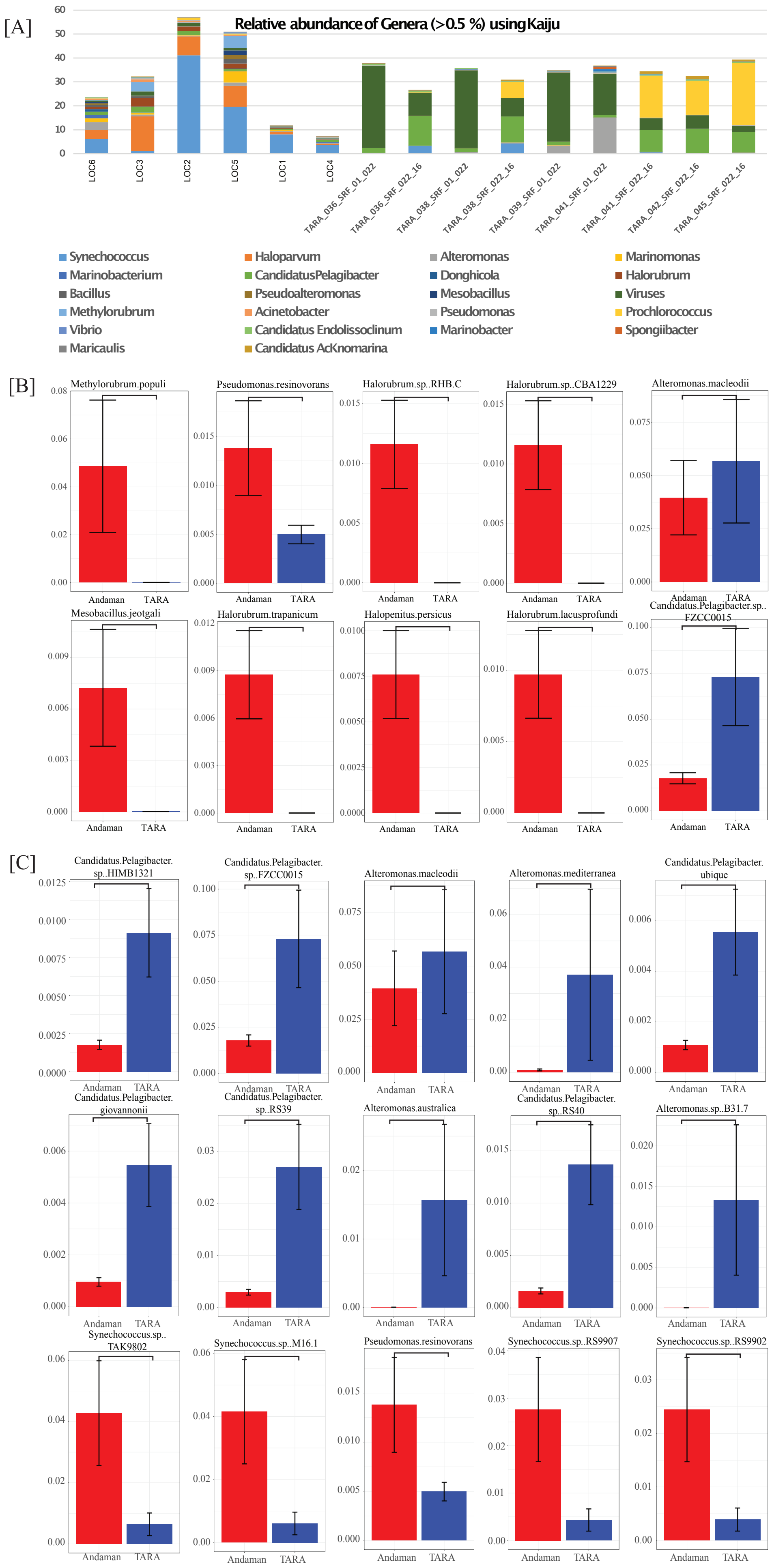

SupplementaryFigure4: **[A]** Relative abundance of microbial genera (with criteria >0.5%) using metagenomic data Andaman and Tara samples. The taxonomic classification of metagenomic reads were carried out using Kaiju. **[B, C]** Microbial species other than Synechococcus and Prochlorococcus (detected using kraken2) among most abundant 20 species in metagenomic data of Coastal (Andaman) and Open-ocean (Tara) samples.

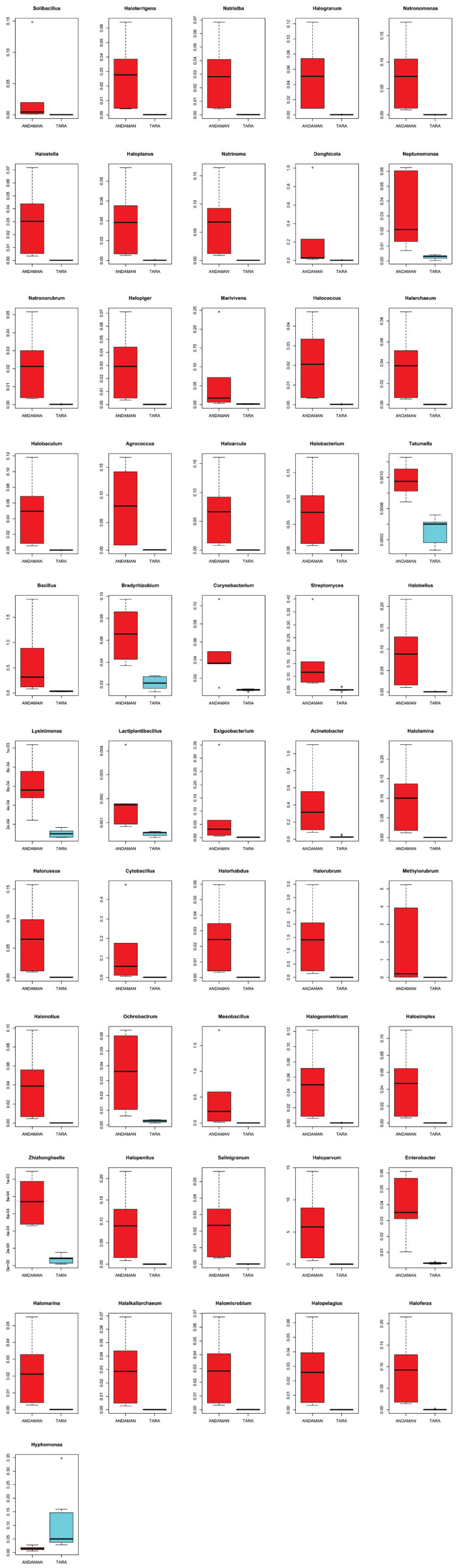

Supplementary Figure 5: Relative abundance of Genera (kaiju) found to be differentially abundant in coastal and Open-ocean samples using LEfSe and Boruta.

[A]

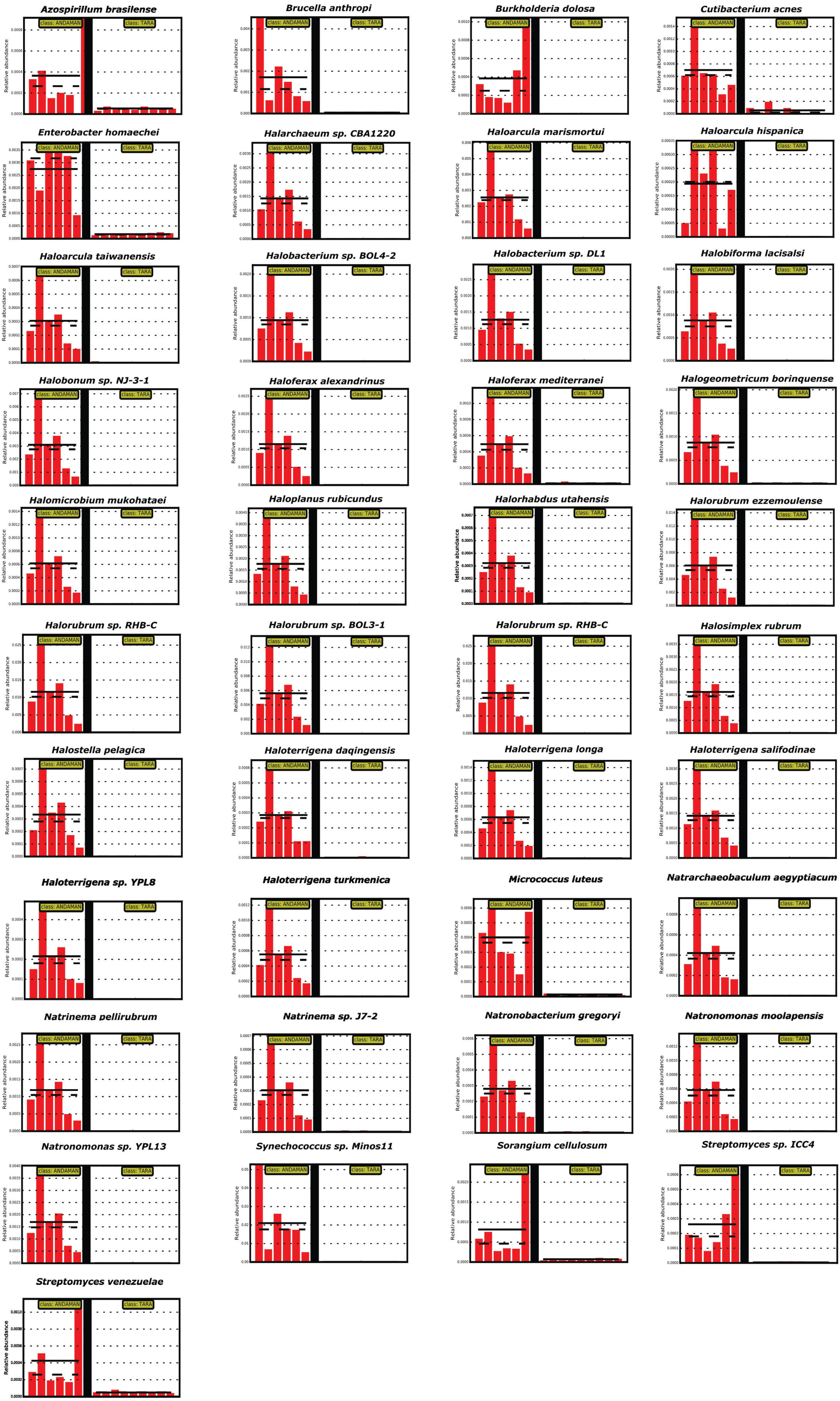

Azospirillum brasilense
Brucella anthropi
Burkholderia dolosa
Cutibacterium acnes
Enterobacter homaechei
Halarchaeum sp. CBA1220
Haloarcula marismortui
Haloarcula hispanica
Haloarcula taiwanensis
Halobacterium sp. BOL4-2
Halobacterium sp. DL1
Halobiforma lacisalsi
Halobonum sp. NJ-3-1
Haloferax alexandrinus
Haloferax mediterranei
Halogeometricum borinquense
Halomicrobium mukohataei
Haloplanus rubicundus
Halorhabdus utahensis
Halorubrum ezzemoulense
Halorubrum sp. RHB-C
Halorubrum sp. BOL3-1
Halorubrum sp. RHB-C
Halosimplex rubrum
Halostella pelagica
Haloterrigena daqingensis
Haloterrigena longa
Haloterrigena salifodinae
Haloterrigena sp. YPL8
Haloterrigena turkmenica
Micrococcus luteus
Natrarchaeobaculum aegyptiacum
Natrinema pellirubrum
Natrinema sp. J7-2
Natronobacterium gregoryi
Natronomonas moolapensis
Natronomonas sp. YPL13
Synechococcus sp. Minos11
Sorangium cellulosum
Streptomyces sp. ICC4
Streptomyces venezuelae
class: ANDAMAN
class: TARA
Relative abundance

[B]

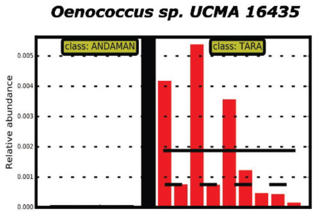

[C]

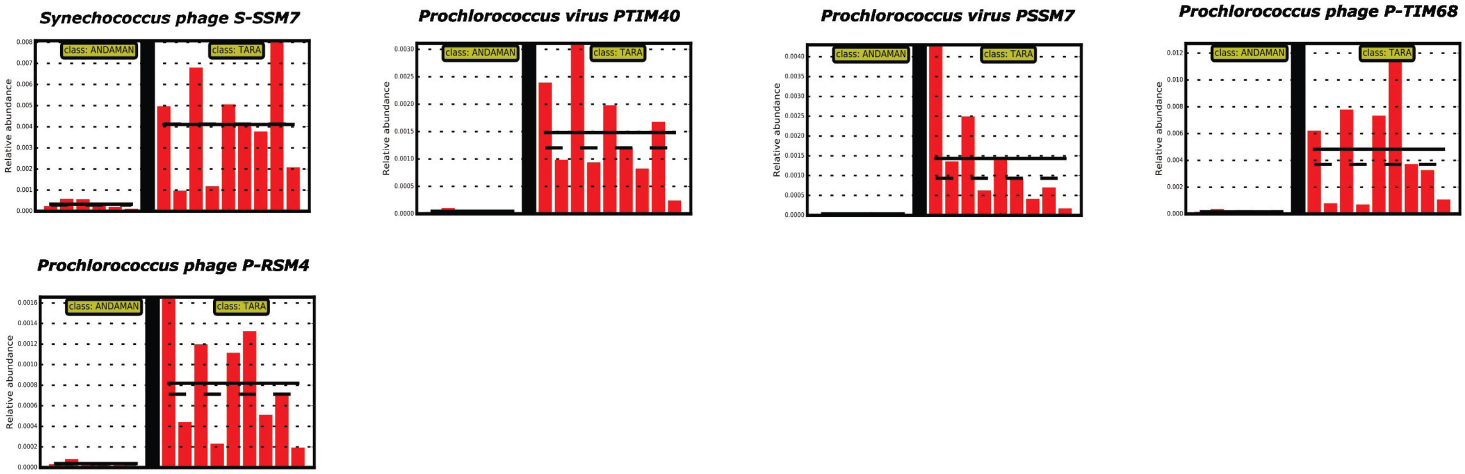

Supplementary Figure 6: Relative abundance of differentially abundant species (kraken2) using LEfSe and Boruta.

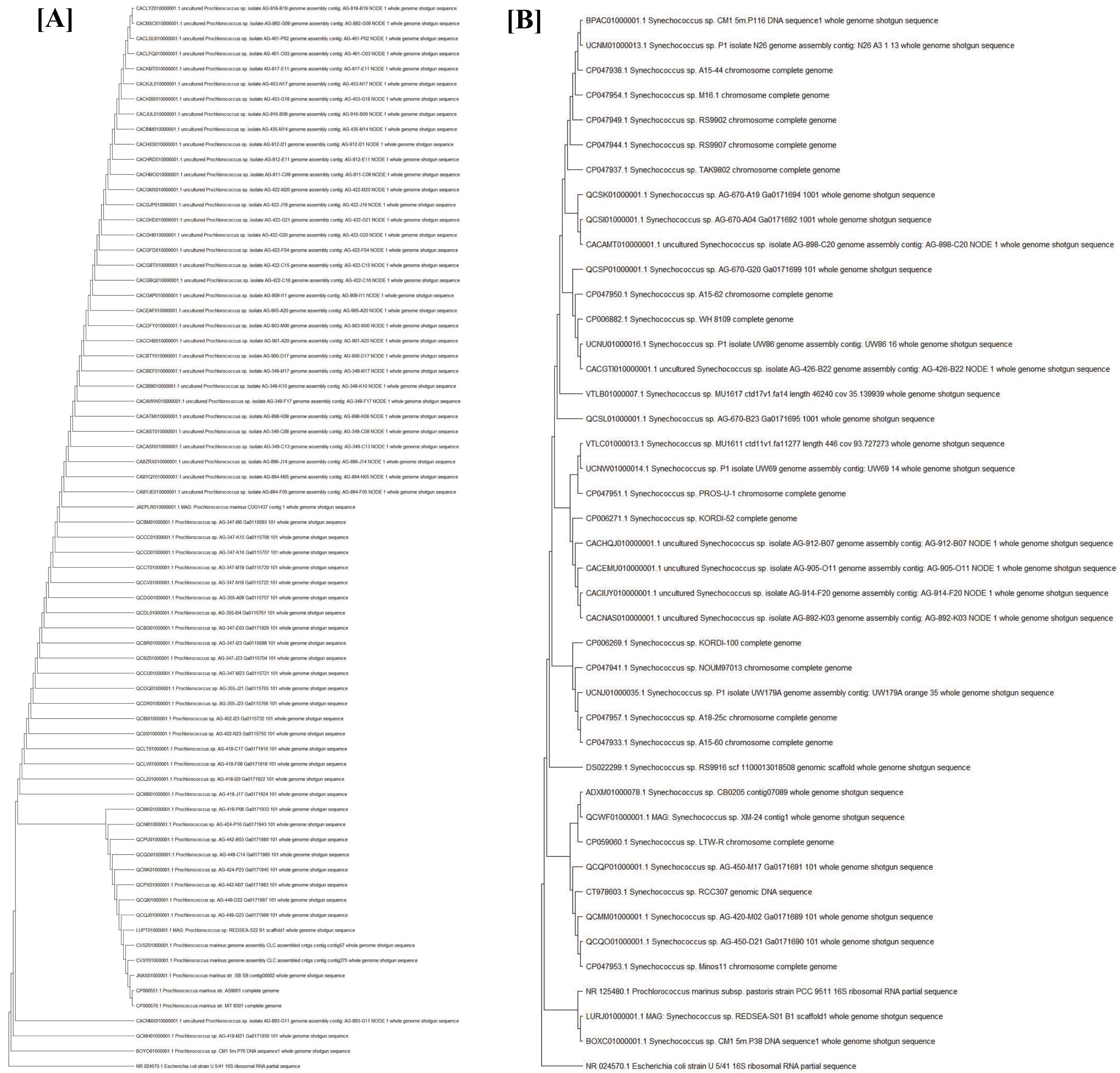

SupplementaryFigure 7: 16S rRNA phylogenetic trees predicted from top abundant **[A]** Prochlorococcus, and **[B]** Synechococcus genomes.
